# Implementing systems thinking and data science in the training of the regenerative medicine workforce

**DOI:** 10.1038/s41536-022-00271-2

**Published:** 2022-12-24

**Authors:** Anne L. Plant, Nicole Piscopo, Krishanu Saha, Claudia Zylberberg, Krishnendu Roy, Katherine Tsokas, Samantha N. Schumm, Sarah H. Beachy

**Affiliations:** 1 Biosystems and Biomaterials Division, National Institute of Standards and Technology, Gaithersburg, MD USA; 2 CRISPR Therapeutics, Cambridge, MA USA; 3 Department of Biomedical Engineering, University of Wisconsin-Madison, Madison, WI USA; 4 Akron Bio, Boca Raton, FL USA; 5 Department of Biomedical Engineering, Georgia Institute of Technology, Atlanta, GA USA; 6 Janssen Research and Development LLC, Spring House, PA USA; 7 Health and Medicine Division, National Academies of Sciences, Engineering, and Medicine, Washington, DC USA

**Keywords:** Regenerative medicine, Medical research

## Abstract

*Applying Systems Thinking to Regenerative Medicine—A Workshop* was organized by the National Academies of Sciences, Engineering, and Medicine’s Forum on Regenerative Medicine. The meeting brought together leaders from government, academia, industry, professional associations, foundations, patient communities, and other stakeholder groups to address the potential for cross-disciplinary systems thinking to advance regenerative medicine. Discussions during the meeting covered the role of data science in regenerative medicine and the importance of data science training and data literacy for the current and future regenerative medicine workforce.

## Introduction

Regenerative Medicine and Advanced Therapies (RMAT) are complex products and often include “living” cellular materials. Every patient—and, for some applications, every product—is unique, and product manufacturing entails lengthy, complicated processes. Intricate relationships exist between the patient’s disease state, the donor starting material, the manufacturing process, supply-chain logistics, and the clinical response. RMAT products are stimulating a paradigm shift in biopharma manufacturing because it means understanding interrelated systems at multiple scales—from molecular and cellular processes to optimization of the manufacturing parameters and workflow, supply-chain logistics, and patient queuing. Therefore, the RMAT enterprise is well-suited to applications of systems thinking, i.e., the use of systems theory and system sciences to analyze relationships and develop effective action in complex contexts. The next-generation workforce can be prepared for this environment with modern data tools and methods and a systems-thinking mindset to extract insights using techniques like data mining and machine learning.

**Fig. 1:**
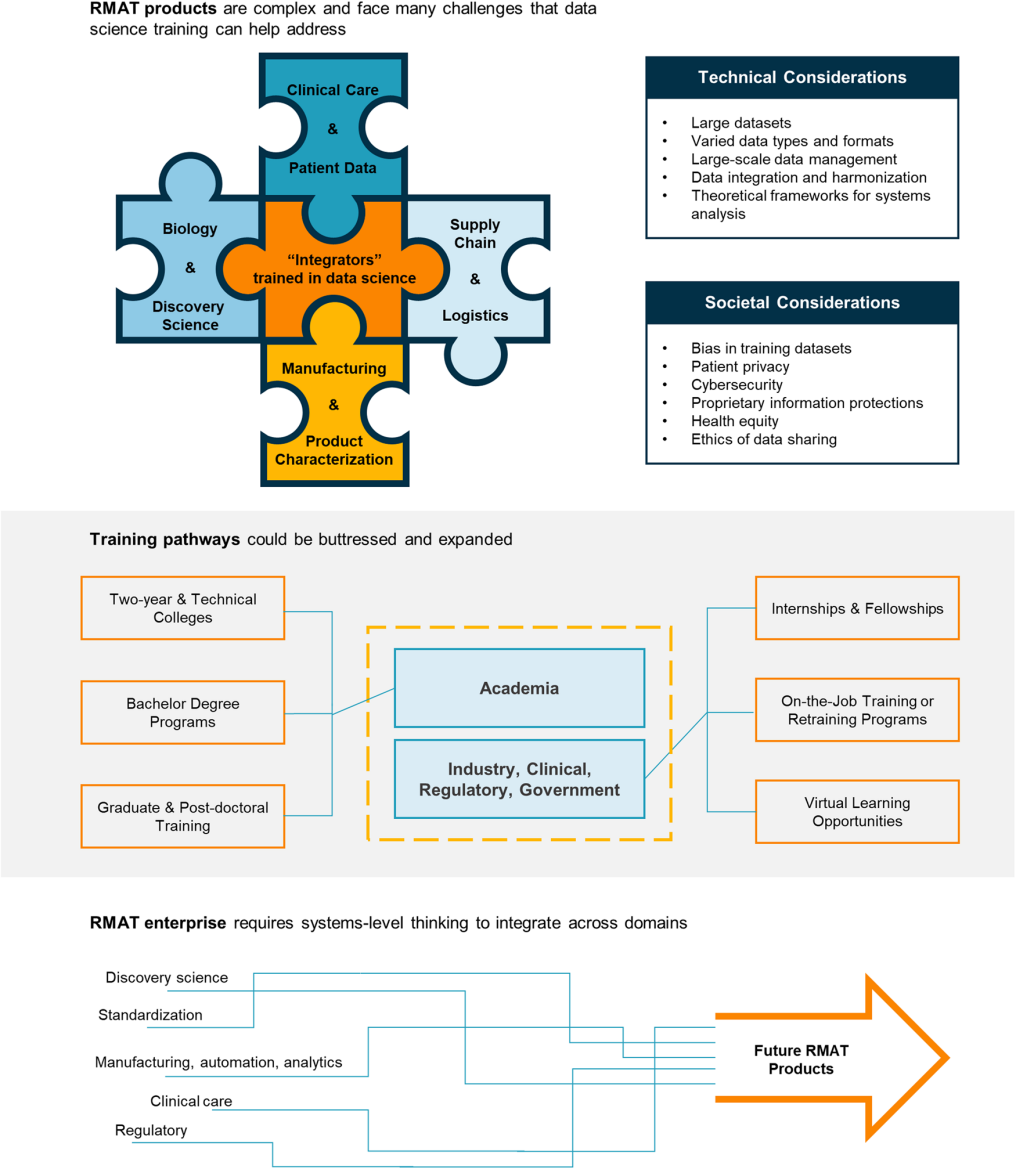
Summary of the role of data science training in the future of RMAT workforce development. The role of data science training in the future of RMAT workforce development. Like a puzzle piece joining parts of a whole, data science can help to connect areas such as clinical care, discovery science, and manufacturing in the field of RMAT. Training can take many forms, including both academic paths and others. Systems-level thinking could enhance the development of new RMAT products.

Cooperation between biologists, engineers, and computational scientists is important to achieving the promise of RMAT products as personalized therapeutics. Systems thinking leverages large, multifaceted datasets to establish reliable manufacturing and distribution methods and to define cellular identities that match intended applications in patients^1^. Data science, data-driven models, and mathematics can link various disciplines, such as stem cell biology, physiology, immunology, and engineering, in effective transdisciplinary research^2^. It would be beneficial to prepare a workforce of “integrators” who can apply sophisticated computational approaches to regenerative medicine research, interpret multidimensional data across sectors of the RMAT enterprise, and translate results of basic research to manufacturing, effective regulations, and the clinic (Fig. 1).

Given the complexity of RMAT products, the Forum on Regenerative Medicine of the National Academies of Sciences, Engineering, and Medicine (NASEM) held a workshop called *Applying Systems Thinking to Regenerative Medicine*^3^. The workshop examined long-standing challenges associated with characterizing patient-specific RMAT therapies to enable successful products, processes, analytics, and supply-chain logistics. Discussions made clear that this field presents significant data-related challenges. The data take many forms, including multi-omics data, functional data, analytical data from processes and manufactured products, supply-chain and healthcare logistics, and patient data. The variety of data requires combining and harmonizing data across sources and formats, and the small size of some first-in-human clinical trials limits analytical power and augments the need for data sharing. These demands create technical challenges like handling, integrating, and interpreting a “system” of large, diverse, and complex datasets, as well as challenges associated with maintaining privacy, cybersecurity, and proprietary information.

Interpretation of data to characterize RMAT products and predict clinical success involves advanced statistical methods and mechanistic models. Advanced quantitative methods may be essential knowledge for the modern technical expert, yet these are not currently a core component of biological research training. Indeed, concepts such as entropy, stochasticity, and critical phenomena are relatively new to cell biology applications^4^. Efforts to integrate process and product analytics with quality control and to integrate manufacturing data with patient-queuing and supply-chain logistics could benefit from systems-level thinking that is currently unprecedented in biopharma and bioengineering. Providing broad-based coursework, hands-on training, and real-life experiences to support the well-versed RMAT practitioner may be challenging but could be advantageous for the clinical and commercial success of the industry. Although not everyone in the RMAT workforce need be a data science and modeling expert, all workers may need a basic understanding of data handling, analytics, and interpretation.

A recent series of NASEM meetings highlighted the intersection of data science with traditional domain sciences and how this interplay impacts data science teaching^5^. Rooted in disciplines such as computer science, statistics, and mathematics, data science is an interdisciplinary field enriched by collaborations between several disciplines. In addition to the technical aspects of data science and applications to specific scientific domains, the societal considerations of data science merit special attention for RMAT practitioners. Societal and ethical issues include how data and communities interact and the role of inclusion and equity in collection, interpretation, and application of data.

In this perspective, we discuss the challenges facing the next-generation RMAT workforce in utilizing data encountered in this field. We consider a wide range of data issues that will be critical for the diverse field of RMAT products. Our goal is to raise awareness of this critical aspect of RMAT for educators and industry professionals. We believe that training the future workforce may occur in traditional and nontraditional ways, and can include 2-year colleges, research universities, graduate and postdoctoral training as well as training opportunities for the existing biopharma workforce.

## Aspects of data science of special relevance to RMAT

Areas of the RMAT development enterprise where data science will play an important role include both technical, scientific issues and related social and ethical issues (Fig. 1).

### Societal and ethical considerations in data science

Artificial intelligence/machine learning (AI/ML) algorithms are increasingly applied to analyze data and make predictions in regenerative medicine^6–9^. As has been widely acknowledged, such algorithms can be biased^6,10^. Since AI/ML strategies contain proxies for true variables of interest, label-choice bias can exist. Recent studies applying AI/ML to healthcare are instructive for the regenerative medicine field because “health”—like “cell quality”—is inherently subjective, holistic, and multidimensional; there is no single, precise way to measure it. A recent study in healthcare, for example, found that bias occurs when an AI/ML algorithm uses health costs as a proxy for health needs^11^. Further, datasets may be biased in terms of demographics like race, gender, or socioeconomic status. Bias can occur during labeling of training data, as a result of how data are collected, or due to self-selection by members of the target populations. For instance, in one study, 87% of patients were white, and most were relatively affluent^12^. The preponderance of data from this demographic may not provide an adequate training set to apply to non-white, non-affluent populations.

To begin addressing these issues, guides were developed about how to define, measure, and mitigate bias in algorithms^10,13^. Privacy, accountability, safety, transparency, fairness and non-discrimination, human control of technology, professional responsibility, and effectiveness are among the key elements^14^. These playbooks and principles could be adapted for regenerative medicine contexts and incorporated into workforce training. Data science training can also prepare the workforce to work with large datasets and leverage other efforts to alleviate health data disparities^15,16^.

### Cybersecurity

The RMAT workforce should be prepared to address issues of cybersecurity. Biotech has been the target of cyberattacks, putting the health of patients in jeopardy^17^. Protecting patient and company data, including intellectual property and sensitive health information, is critical and challenging. Cybersecurity is a critical feature of establishing a robust RMAT supply chain. The National Institute of Standards and Technology (NIST) defined a framework for cybersecurity in 2018 and has since updated guidance^18,19^. The framework provides general guidance for assessing cybersecurity risk, managing cybersecurity within the supply chain, and reducing risk to critical infrastructure. Again, adapting this framework for regenerative medicine could help assure secure RMAT supply chains in accord with federal and institutional standards.

### Data sharing and data integration

A systems-based approach to solving the complex challenges faced in regenerative medicine requires data sharing across stakeholders, and the up-and-coming workforce can be instrumental in achieving that aim. Although encouraged by some funding agencies, including the National Institutes of Health (NIH)^20–22^, widespread data sharing has been uncommon to-date in cell biology, clinical research, and manufacturing. Data sharing can advance regenerative medicine by enabling studies with greater statistical power, a wider range of variables, and a more diverse patient base than single studies can achieve. Collaboration can harness new and existing data sources to promote data-driven scientific discovery and innovation across the private and public sectors globally^23,24^. Incorporating FAIR Guiding Principles for scientific data (Findability, Accessibility, Interoperability, Reuse of digital assets) in research practices and workforce training encourages open access and furthers the public good^25^. Such approaches can provide advantages for RMAT akin to those from the Bermuda Principles, which enabled the Human Genome Project to share data shortly after sequencing and strengthened international cooperation^26,27^.

Data sharing also facilitates comparability studies and the development of standards for RMAT products. Reproducibility remains a challenge in RMAT research and product development and is largely attributable to complex and highly manual manufacturing processes, variability in donor and patient cells, reagent quality variability, and the lack of standards and comparability metrics. Data sharing and standardization could alleviate these issues and significantly improve outcomes in trials with RMAT products. Finally, the regenerative medicine workforce should be cognizant of the legal challenges of data sharing related to intellectual property and how data sharing may impact the security of patient data^28,29^. Data sharing could substantially advance the field but remains contingent on appropriate data management and security practices; creating such infrastructure is ongoing^30^.

## Data science education pathways

Education pathways must continue to evolve so that students are data-literate and prepared for careers in domains like regenerative medicine. Integration of data science perspectives into data-intensive domain courses can yield transformative researchers and practitioners educated in both the foundations and domain-specific applications of data science. Integrating data science education throughout the RMAT training pipeline can assure that students are versed in the language of data collection, analysis, interpretation, management, and security.

Implementation of data literacy programs ensures a critical mass of workforce talent. Indeed, many universities have launched data science institutes, programs, or schools. The 2018 Moore-Sloan Foundation’s report on the status of data science highlights approaches to data science at universities. Approaches vary across investment strategy (e.g., campus and private funding), curricular emphasis (e.g., engineering, information/computer sciences, business, mathematics/statistics), and organization (e.g., institute, school, department/division)^31^.

### Undergraduate education

Many institutions recognize the increasing role of data in the workplace and the commensurate importance of data literacy for new graduates. Attempts to meet the need for data science training range from specialized coursework housed in existing statistics or computer science departments to new programs and majors, such as the Data Theory Major at the University of California, Los Angeles^32^. A 2018 NASEM report details various routes for data science training at the undergraduate level^33^, yet training opportunities could be expanded.

Competencies for these programs comprise data science foundations and applications and may incorporate ethics, legal and regulatory issues, and social concerns. Flexibility and incentives for sharing courses, materials, and faculty would support nascent programs. A key challenge of incorporating additional data science courses in undergraduate curricula is the constraint of not increasing the total credit hours for degree programs—i.e., another course must be removed to accommodate the data science course(s). Gaining consensus among programs on reallocating course content would be challenging, but doable, and advantageous. An alternative or complementary approach could be adding data science training and projects to existing courses with domain-specific examples.

### Graduate education

Graduate education could include innovative programs in data science, delivered through modular and nontraditional methods, including applying asynchronous pedagogical strategies to residential and non-residential digital learning. Masters and doctoral students could enroll in data science programs or domain-specific programs with a data science certificate. Possibilities include modular, stackable graduate certificate programs and three graduate degrees in data science: (i) professional science masters, (ii) masters, and (iii) doctoral.

Graduates of data science programs work in all job sectors and serve in many roles. Although not everyone in the RMAT workforce needs data science expertise, most workers—across all levels—should understand the language of data science well enough to interpret analysis of RMAT products and to communicate with data science specialists. Students will acquire data science literacy through exposure to data science throughout the graduate and undergraduate curriculum.

### Complementary opportunities for data science education

Complementary educational pathways could fill gaps in the RMAT training pipeline. Community or technical colleges are one avenue for providing data science training and guiding individuals into regenerative medicine. Two-year colleges are already developing courses, certificates, and associate degrees in data science, analytics, and data management to meet the demand for a data-literate workforce^5^. For example, the Northeast Wisconsin Technical College offers a Data Analytics Associate’s Degree to prepare students to work in fields like advanced manufacturing and healthcare. Through partnerships with 4-year colleges and national or regional centers focused on biomanufacturing or data sciences (e.g., BiofabUSA, BioMADE), biotechnology programs at 2-year colleges can offer experiential, project-based learning and enhanced training opportunities for entry-level workforce. For example, the National Science Foundation Engineering Research Center for Cell Manufacturing Technologies (CMaT) offers hands-on research and training opportunities for 2-year college students in Georgia and Wisconsin and works with 2-year college instructors to develop curriculum.

Data science boot camps, which are intensive skill-building programs, can complement other academic or industry experience and better connect participants with industry demands^5^. Internships can also supplement the education of new graduates from traditional academic programs who may lack industry experience. Experienced employees might benefit from continuing education programs that leverage and repurpose the on-the-job workforce^5,34^. The future of workforce development will also include educational opportunities outside of academic institutions, and online workshops, webinars, and courses could be further expanded and supported by various sectors of the regenerative medicine field. Moreover, virtual learning opportunities may be accessed anywhere by individuals with varying levels of experience and education, and asynchronous options may be accessed anytime, thus supporting diversity, equity, and inclusion in the workforce. An example is a recent 12-lecture virtual and asynchronous course launched jointly by the International Society for Cell and Gene Therapy (ISCT) and CMaT; the course covers concepts in regenerative medicine and cell manufacturing, is open to anyone around the world, and is co-taught by academic and industry leaders^35^.

## Creating synergy through multisector involvement

The demand for a data-literate workforce creates a need for synergy between stakeholders across sectors (e.g., industry, academia, and government) of the RMAT enterprise. In academic settings, curricula to prepare data scientists for work in regenerative medicine fields might include topics in regulatory affairs, clinical development, and manufacturing. These topics will equip students to understand the unique context of regenerative medicine and appreciate systems thinking. Academia can also bolster the RMAT ecosystem by increasing the knowledge base of educators and providing resources for students interested in nonacademic careers. Synergy occurs from sectors working together on key areas of interest and providing both formal and informal educational opportunities that bridge data science and regenerative medicine. The organizations mentioned below are examples of strong multisector activities that can play an effective role in developing the RMAT workforce.

### Regulatory

Workforce development in data-dependent regulatory issues warrants special attention. Because RMAT products are highly variable and customizable, the regulatory landscape continually evolves, and the regulatory sector must keep pace with advancements. To facilitate crosstalk and opportunities to use data science for regulatory decision-making, the regulatory affairs workforce needs to understand data science fundamentals, and the data science workforce needs a basic understanding of regulatory issues. Now is a key time to train a data science workforce to manage the regulatory environment of a rapidly evolving product and application space.

Regulatory training can begin through academic courses and degree programs^36^. The U.S. Food and Drug Administration (FDA) interfaces with several academic institutions within the Centers of Excellence in Regulatory Science and Innovation (CERSI) program to help train students in regulatory science^37^. However, regulatory instruction could also be incorporated into existing courses. Reallocating modest portions of curricula across multiple academic stages could better prepare students to interact with regulatory guidance and to prepare documents for regulatory agencies. Statistics courses could train students to analyze large datasets with the goal of introducing regulatory concepts such as critical quality attributes (CQAs), critical process parameters (CPPs), normal operating range (NOR), and proven acceptable range (PAR). Lab-based courses could teach students to prepare process descriptions in accordance with FDA guidance documents and to describe theoretical process-characterization strategies based on risk-assessment exercises conducted in class. Advanced courses could encourage students to discuss the applicability of data science approaches (e.g., real-world data, digital transformation, AI/ML) for regulatory decision-making through answering questions such as:

- How can real-world data address regulatory filing requirements from clinical, preclinical, and manufacturing perspectives?
- How can data science tools help establish the safety and efficacy of a regenerative medicine product?

### Clinical and translational science

Discoveries in stem cell biology and associated technologies often occur in basic science laboratories, but the promise of regenerative medicine is realized in the clinic. Academia can prepare students with interest in clinically oriented roles by emphasizing data science and theoretical analysis in programs like the Institute for Clinical and Translational Research (ICTR) at the University of Wisconsin–Madison, which offers minors in Clinical Investigation for PhD students, or the Georgia Clinical & Translational Science Alliance (CTSA), an NIH-funded program across Georgia-based universities that offer a Master of Science in Clinical Research and certificate program in translational research for PhD trainees. Such programs encourage students to consider the human impact of scientific discoveries and promote translational research.

Ranging from data acquisition and harmonization to patient privacy, data-related challenges can impede the translation of regenerative medicine discoveries to the clinic. Small sample sizes often limit research in the rare disease space, and data integration among research groups and datasets can hamper progress. To contend with these issues, several institutions established their own data-sharing capabilities, including Johns Hopkins, the Mayo Clinic^38^, CMaT^39^, and others. CMaT, for example, works to standardize methods across its eight-university ecosystem and partners with companies to record data in a unified format via batch recording software. Data can be stored in the cloud for collaborators across partner organizations to access and analyze.

Started by nine medical research organizations, another initiative, the National Center for Data to Health (CD2H), facilitates data sharing and collaboration across the community of health informatics researchers^34^. The National Heart, Lung, and Blood Institute (NHLBI), including the NIH-wide Regenerative Medicine Innovation Project (RMIP)^40^, also established a data-sharing platform for NIH-funded projects—the Biodata Catalyst—which is intended to serve as a central data repository for open sharing among eligible researchers^20^. Moreover, a workforce that can manage, interpret, and deploy data science efficiently and securely will allow the RMAT industry to capitalize on potential benefits, both nationally and globally^41^.

### Manufacturing

As the RMAT industry grows and new, increasingly complex products enter the pipeline, highly trained and appropriately certified workers will be in-demand to manufacture high-quality products, at scale, with low batch failure rates and maximal reproducibility—all while ensuring efficacy and patient safety and maintaining strict regulatory standards. Biomanufacturing is increasingly digital and therefore requires proficiency in both quantitative methods and biology-based lab techniques. Moreover, a potential future model of manufacturing is decentralized, remote manufacturing in hospital settings. To support this scheme and other distributed manufacturing approaches in the future, understanding remote access, digital networks, and AI would benefit the workforce.

Academia can leverage guidance documents designed for industry to better prepare students for roles in manufacturing regenerative medicines^42^. When designing curricula, academic entities could integrate discussions with industry and clinical manufacturers to better understand the skillsets needed for biomanufacturing. Manufacturing sciences as a separate discipline is only available at a few academic centers; rather, manufacturing is often taught through mechanical or chemical engineering departments. Collaboration between experts in manufacturing sciences and other specialized domains (e.g., cell therapy, biofabrication) would help ensure curricula and training materials reflect domain knowledge in the context of broader manufacturing principles. This collaborative, transdisciplinary effort across engineering, cell biology, clinical translation, and industrial manufacturing will be critical to prepare the next generation of RMAT workforce.

Beyond educational efforts, academic scientists can better coordinate with industry by considering manufacturing guidelines, such as Good Manufacturing Practice (GMP) regulations, in their research. To facilitate translation from bench to bedside and increase uptake of innovations in the clinic, academic laboratories and researchers could aim to develop new devices, tools, software, and technologies that are GMP-compatible, follow Quality-by-Design principles, incorporate standardized analytical tools and measurements, and implement regulatory constraints.

Finally, key technology hubs and public-private partnerships play an important role in integrating data knowledge into workforce development. Organizations focused on RMAT manufacturing include the aforementioned CMaT, Marcus Center for Therapeutic Cell Characterization and Manufacturing (MC3M), National Institute for Innovation in Manufacturing Biopharmaceuticals (NIIMBL), BioFabUSA by the Advanced Regenerative Manufacturing Institute (ARMI), the Catapult Network in the UK, and the Centre for Commercialization of Regenerative Medicine (CCRM) in Canada. Examples of important steps these organizations have taken to build a dynamic workforce include:

- Collaborative course module development for both technical and ethics/regulatory competencies.
- Collaborations with 2-year college systems for hands-on training and curriculum development.
- NSF-funded Future Manufacturing Network (FMNet) Consortium for “Building a Network to create the Workforce Foundation, Actionable Roadmap, and Infrastructure Design to Integrate Data Science, AI, and Predictive Analytics throughout Biomanufacturing”.

Notably, most of these efforts are in the early stages and need investment to scale up nationally or internationally. Community-based, distributed workforce training programs that build industry-identified skillsets and incorporate robust certification could significantly advance the successful use of large-scale data in regenerative medicine.

## Conclusions

The rapidly evolving field of RMAT requires a workforce trained to take full advantage of data from different sources and at scale. Addressing the challenges of the complex systems that define the RMAT enterprise may entail forming large, unbiased datasets for statistically meaningful analysis. A workforce equipped with both the scientific and technical knowledge and the collaboration skills to integrate expertise can move the field forward in all aspects of regenerative medicine, including basic research, regulation, and manufacturing.

To deliver on the promise of regenerative medicine relies on the field to work together across sectors to train the future workforce to leverage modern tools of data science and facilitate the success of RMAT products as science and technology continue to progress. This paper is a call to bring attention to the integration of data science and the application of systems thinking for addressing the challenges faced in developing regenerative medicines.

## Data Availability

Data sharing is not applicable to this article type, as no datasets were generated or analyzed.
